# Supplementary figures and images for: Novel sampling methods for monitoring Anopheles arabiensis from Eritrea
Source: PeerJ. 2021 Jul 19;9:e11497. doi: 10.7717/peerj.11497 (PMC8297471; doi:10.7717/peerj.11497)

16

17

18

19

20

21

22

23

24

25

26

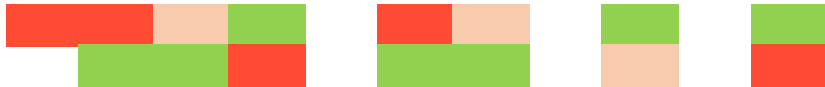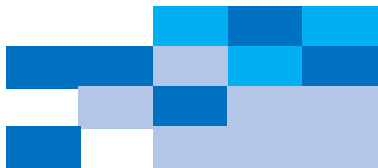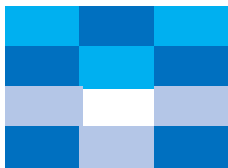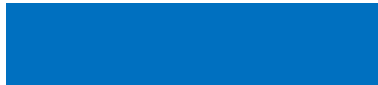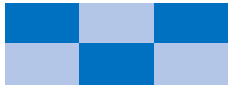

Supplement: Supplemental Information 2 — Collection type by date and location. Further Suna trap collections were performed in other locations (1 collection in the village and 2 close to the sentinel trap) without result. [file peerj-09-11497-s002.pdf]
