# Supplementary material for: Novel sampling methods for monitoring Anopheles arabiensis from Eritrea
Source: PeerJ. 2021 Jul 19;9:e11497. doi: 10.7717/peerj.11497 (PMC8297471; doi:10.7717/peerj.11497)
Supplement: Supplemental Information 3 [file peerj-09-11497-s003.docx]

**Exploratory Data Analysis**

**Mosquito counts by collection methods**

Figure 1 suggests that in general, the number of mosquitoes collected at Site 1 was considerably high compared to Site2, and that both Backpack and Procopack have produced great number of mosquito compared to aspirator.


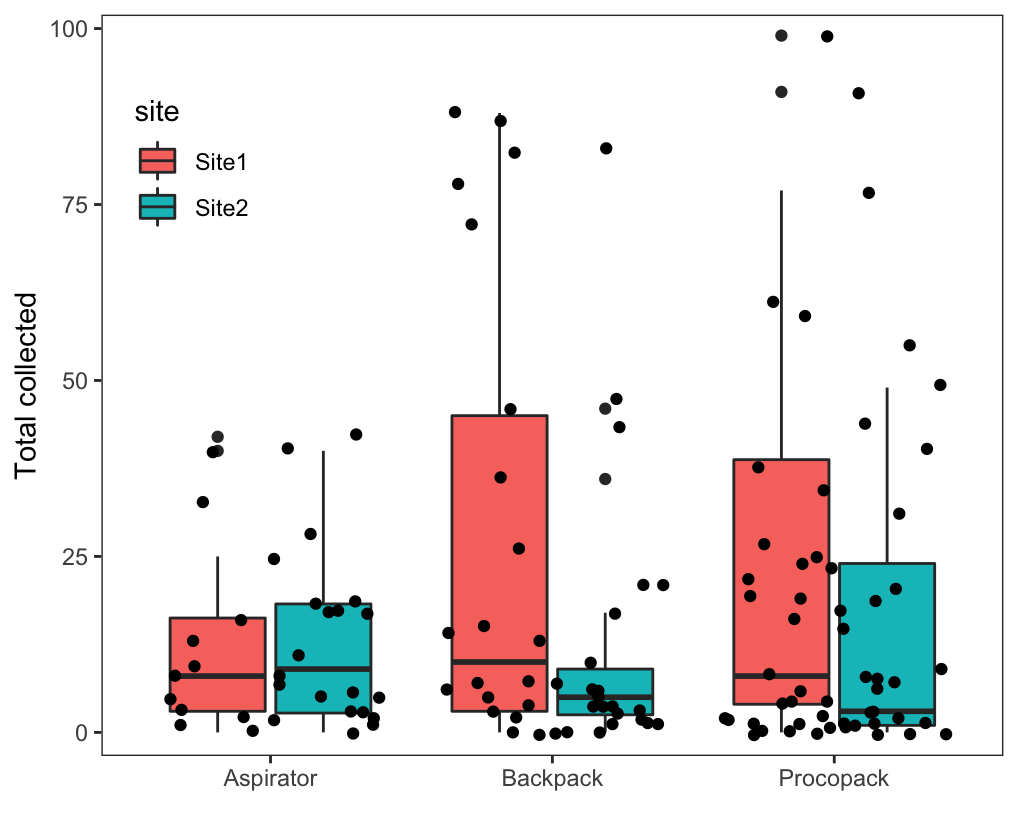


**Figure 1.** Variation of mosquito catches obtained by different collection methods according to study site

**Influence of collector and site**

Figures 2A shows that not all participants performed collection using the three methods and also there was relatively great variability in the size of mosquito catches obtained by each collection method both between and with collectors. Moreover, there is indication of some variation of mosquito catches size among collectors and methods across sites (Fig 3B).


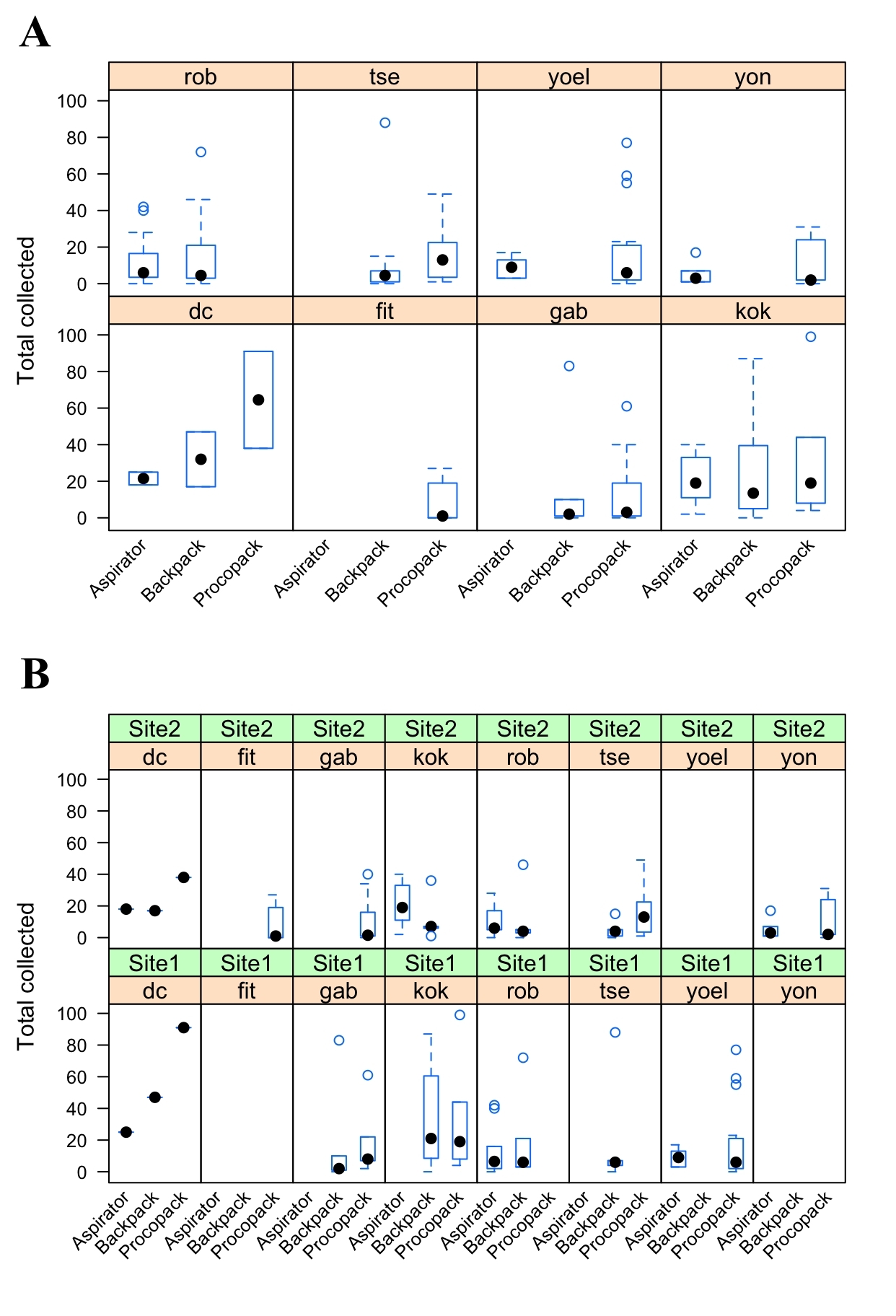


**Figure 2**. Relationship between collector (A) and the interaction effect collector: site (B) on mosquito catch

**Sampling regime and collection performance**

Data show a sharp reduction of mosquito counts after the first sampling round, irrespective of collection methods and sites. The data also suggest that Procopack performed better compared to other methods, although at site 2 the three methods produced consistently low mosquito counts compared to site 1. It worth noting that, the performance of all methods seems to be comparable after the second collection regime.

**Figure 3.** Relationship between collection methods, site and sampling regimes on mosquito catch

**Statistical analysis**

The density distributions of mosquito counts obtained by the three methods are heavily (right) skewed (supplementary Fig. 1S(A)), which suggests that mosquito counts may be over-dispersed and, thereby, plausibly described by a negative binomial distribution function. Additionally, collections were repeatedly performed by same individuals and at same places (site 1 and site 2) over study period. These repeated measurements have inevitably introduced some amount of dependency (correlation structure) between observations (supplementary Fig. 1S(B)). As such, Generalized Linear Multilevel Models (GLMMs) with negative binomial error distribution and log-link functions were applied to model the difference in sampling catches produced by the three collection methods, that is, Aspirator, Backpack and Procopack. Collection methods (a three levels factor: Aspirator, Backpack and Procopack), site (binary factor: Site 1 and Site 2) and collection regimes (five levels factor: 1^st^ – 5^th^ rounds) were considered as fixed factors. To accommodate potential correlation and explicitly nesting structure of repeated measurements across collectors and along study period, as evidenced by aforementioned exploratory analysis, both collector and time (in days) effects were modelled as crossed random factors. This allowed the performance of each collection methods to vary among collectors and days within sites. Model fit was assessed by visual inspection of the graph of standardized model residuals against fitted observations [1]. The amount variation of mosquito counts explained by predictors was determined by both marginal and conditional coefficient of determination *R^2^,* proposed in [2]. Additionally, Akaike Information Criterion test (AICc) was also applied to guide the selection of the best model fit. The most parsimonious model (the one with the lowest AICc) was preferred among the others. Final estimation of the mean and 95% Credible Intervals bands (95% CrI) of fixed effects coefficients were obtained via simulations from posterior distribution of best model fit parameters. A total of 2000 random simulations were performed using the function sim of the arm packages [3]. The performance of Backpack and Procopack in relation to Aspirator was estimated in terms of Incidence Rate Ratio (IRR). All the data processing tasks and statistical analysis were performed using the R software version 4.0.2 [4].

**Results**

The model fit (AICc = 852.2) was the one that included collection methods, site as main effects factors and also the interaction effect between collection methods and sampling regimes. The intercepts of mosquito counts varied between collectors and study period within sites. Both fixed and random factors explained 86.6% of the variability in mosquito counts (Tab 1). Table 1 and Figure 2 summarize the results collection methods performance, expressed in terms of IRR (± 95%CrI). Results suggest that mosquito population was, respectively, 1.45 (0.89 – 2.32) and 1.83 (1.17 – 2.29) more likely of being sampled by Backpack and Procopack compared to Aspirator. However, the difference was statistically significant only between Aspirator vs Procopack. No significant difference was detected between Aspirator vs Backpack (Tab. 1). The results also showed that the likelihood of collecting any mosquito significantly between sites, that is, the chance of collecting at Site 2 was 41.6% lower compared to Site 1. There were also significant interaction effects between collection methods and sampling regimes. The risk of mosquito being sampled using all the three methods reduced significantly from the second sampling round to fifth round compared to the first sampling round. For instance, the chance of a mosquito being sampled by aspirator reduced from 47.6% to 95.6%, whereas by Backpack and Procopack it reduced, from 77.2% to 94% and 59.2% to 96.5%, respectively (Tab. 1). The data also suggest that likelihood of mosquito collection by any methods become relatively comparable after the second sampling round, as evidenced by the overlaps of posterior means credible intervals summarized in Fig. 4 and predicted catches in Fig. 5.

**Table 1**. Posterior mean Incidence Rate Ratio (IRR ± Credible Interval bands) of the risk of mosquito collection using Backpack and Procopack compared to mouth Aspirator (reference method). The magnitude of interaction effect between collection methods and sampling regimes is also reported. For sampling regime factor, the first level (i.e., round 1) was considered as reference level. Marginal R^2^ indicates the amount of variance explained by fixed factors only whereas Conditional R^2^ shows the variance explained by both fixed and random factors.

| **Variables** | **Incidence Rate Ratios (IRR)** | **95% Credible Interval** |
| --- | --- | --- |
| Intercept | 38.79 | 24.53 – 60.67 |
| Backpack | 1.45 | 0.89 – 2.32 |
| Procopack | 1.83 | 1.17 – 2.92 |
| Site [2] | 0.58 | 0.46 – 0.73 |
| Aspirator : Round[2] | 0.52 | 0.32 – 0.84 |
| Backpack : Round[2] | 0.23 | 0.15 – 0.34 |
| Procopack : Round[2] | 0.41 | 0.29 – 0.57 |
| Aspirator : Round[3] | 0.25 | 0.15 – 0.41 |
| Backpack : Round[3] | 0.09 | 0.06 – 0.14 |
| Procopack : Round[3] | 0.13 | 0.09 – 0.19 |
| Aspirator : Round[4] | 0.18 | 0.10 – 0.31 |
| Backpack : Round[4] | 0.07 | 0.05 – 0.12 |
| Procopack : Round[4] | 0.05 | 0.03 – 0.08 |
| Aspirator : Round[5] | 0.04 | 0.02 – 0.09 |
| Backpack : Round[5] | 0.06 | 0.03 – 0.10 |
| Procopack : Round[5] | 0.04 | 0.02 – 0.06 |
| **Random Effects** |  |  |
| σ^2^ | 0.25 |  |
| τ_00_ _DayID_ | 0.06 |  |
| τ_00_ _collectorID_ | 0.12 |  |
| ICC | 0.42 |  |
| Marginal R^2^ / Conditional R^2^ | 0.770 / 0.866 |  |


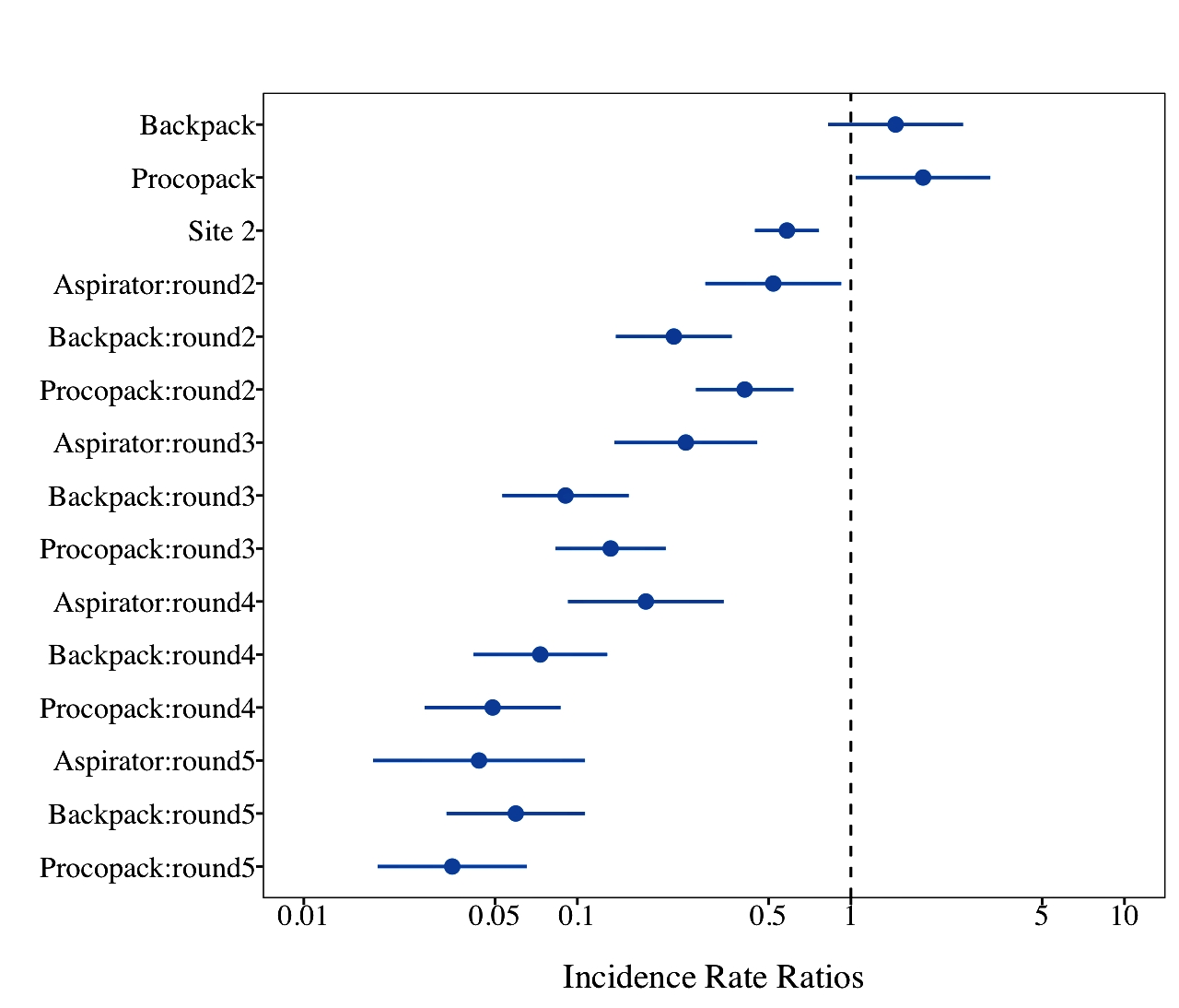


**Figure 4**. Forest plot showing the risk of mosquito being caught by different methods and the magnitude of the influence of sampling regime on collection methods.

***Prediction of mosquito counts***

Figure 5 depicts the result of predicted mean mosquito counts obtained by the three sampling devices between sites and sampling regimes. Results show that the three methods tend to produce the nearly similar size of catch after from second round onward.

**Figure 5**. Predicted posterior mean mosquito counts obtained by different collection methods according to site and sampling regime. Error bars represent 95% credible intervals bands (see methods for further details)

**Supplementary information**

***Distribution of mosquito counts and autocorrelation***

Mosquito distribution heavily tailed and showing temporal autocorrelation over time which is a clear indication of overdispersion and dependence between mosquito counts


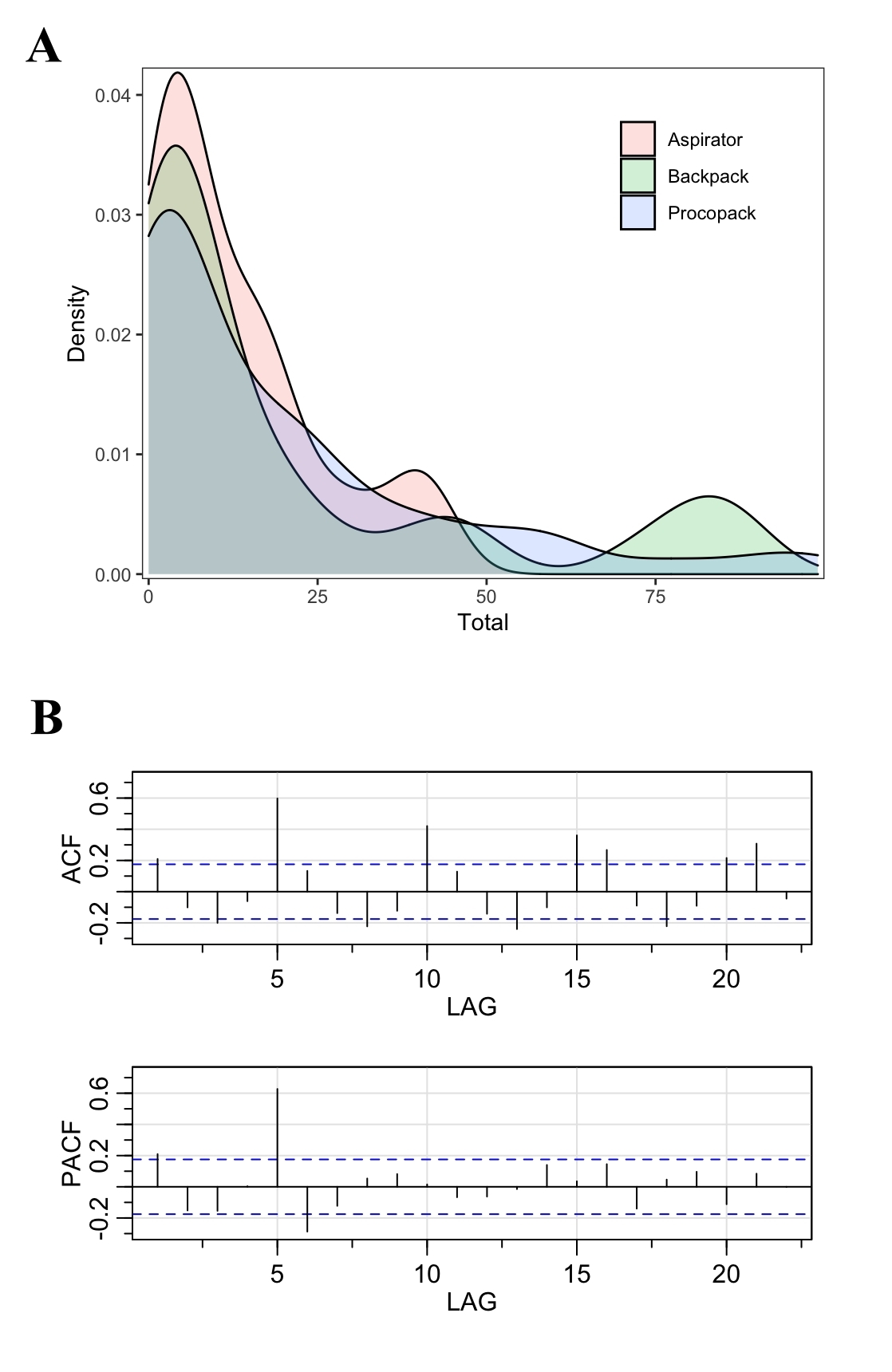


**Figure 1S.** Density distribution of mosquito counts from different collection methods (A) and the temporal dependence of mosquito catches (B). Lags in days.

***Checking model assumptions***

The relationship between expected residuals obtained via empirical distribution of mosquito counts and those obtained by distribution of observed is depicted by the qqplot. The was not significant deviation of observed from expected residual, as evidenced by Kolmogorov-Smirnov (KS) statistics and the distribution of points closer to red line. However, the scatter plot of the standardized residuals against the predicted values indicated the presence of some sort of patterns, although still not significant. Moreover, the mean distribution of random factors residual, that is, collector and Day, was close to zero, that is, 0.0037 and 0.0010, respectively, which is acceptable. The residuals dispersion test was also not significant.


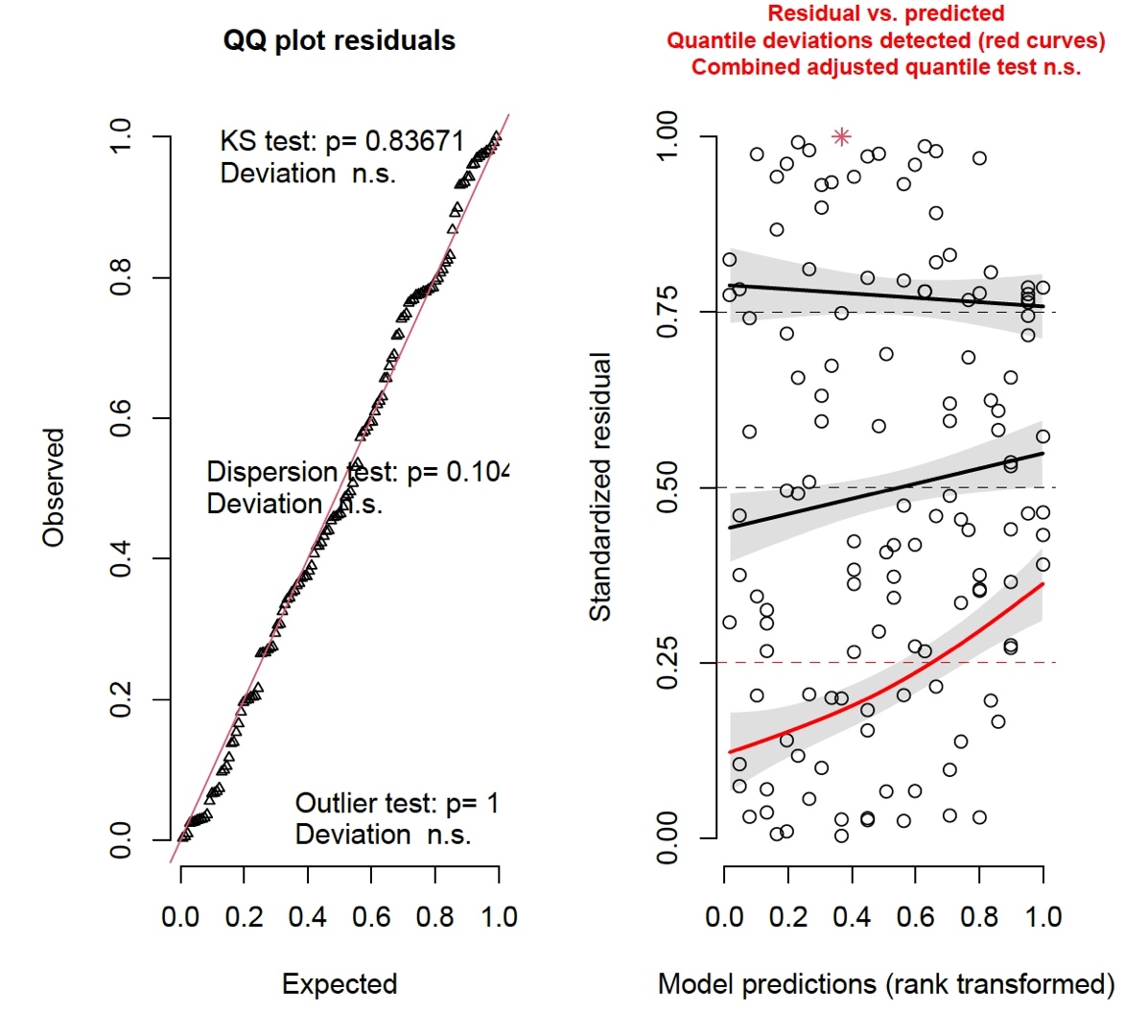


**Figure 2S.** Quantile-quantile plot of residuals from expected counts against observed counts (left) suggest good fit model to observed mosquito counts. However, the relationship between standardized residuals vs predicted counts still suggest some focal deviations (indicated by the red curve on the right panel plot) but the overall deviation was not significant (n.s).

# Bibliography

1. Hartig F: DHARMa: Residual Diagnostics for Hierarchical (Multi-Level / Mixed) Regression Models. *R package version 0320* 2020.

2. Nakagawa S, Schielzeth H: A general and simple method for obtaining R2 from generalized linear mixed-effects models. *Methods Ecol and Evol* 2013, 4:133-142.

3. Gelman A, Yu-Sung S: arm: Data Analysis Using Regression and Multilevel/Hierarchical Models. *R package version 110-1* 2018.

4. R Core Team: **R: A language and environment for statistical computing.** Vienna, Austria: R Foundation for Statistical Computing; 2020.
